# Safety of living liver donors with remnant liver volume below 30% after laparoscopic living donor hepatectomy

**DOI:** 10.1093/bjsopen/zrag116

**Published:** 2026-11-13

**Authors:** Sukmin Gwon, Namkee Oh, Sang Ah Chi, Sunghae Park, Eunjin Lee, Youngju Ryu, Jiyoung Baik, Hayeon Do, Jaehyun Kwon, Hansang Park, Inho Lee, Yoonsun Choi, Jinsoo Rhu, Kyunga Kim, Jongman Kim, Gyu-Seong Choi

**Affiliations:** Department of Surgery, Samsung Medical Centre, Sungkyunkwan University School of Medicine, Seoul, Republic of Korea; Department of Surgery, Samsung Medical Centre, Sungkyunkwan University School of Medicine, Seoul, Republic of Korea; Biomedical Statistics Centre, Research Institute for Future Medicine, Samsung Medical Centre, Seoul, Republic of Korea; Department of Surgery, Samsung Medical Centre, Sungkyunkwan University School of Medicine, Seoul, Republic of Korea; Department of Surgery, Samsung Medical Centre, Sungkyunkwan University School of Medicine, Seoul, Republic of Korea; Department of Surgery, Samsung Medical Centre, Sungkyunkwan University School of Medicine, Seoul, Republic of Korea; Department of Surgery, Samsung Medical Centre, Sungkyunkwan University School of Medicine, Seoul, Republic of Korea; Department of Surgery, Samsung Medical Centre, Sungkyunkwan University School of Medicine, Seoul, Republic of Korea; Department of Surgery, Samsung Medical Centre, Sungkyunkwan University School of Medicine, Seoul, Republic of Korea; Department of Surgery, Samsung Medical Centre, Sungkyunkwan University School of Medicine, Seoul, Republic of Korea; Department of Surgery, Samsung Medical Centre, Sungkyunkwan University School of Medicine, Seoul, Republic of Korea; Department of Surgery, Samsung Medical Centre, Sungkyunkwan University School of Medicine, Seoul, Republic of Korea; Department of Surgery, Samsung Medical Centre, Sungkyunkwan University School of Medicine, Seoul, Republic of Korea; Biomedical Statistics Centre, Research Institute for Future Medicine, Samsung Medical Centre, Seoul, Republic of Korea; Department of Surgery, Samsung Medical Centre, Sungkyunkwan University School of Medicine, Seoul, Republic of Korea; Department of Surgery, Samsung Medical Centre, Sungkyunkwan University School of Medicine, Seoul, Republic of Korea

## Abstract

**Background:**

Donor safety is paramount in living-donor liver transplantation. A remnant liver volume (RLV) ≥ 30% is widely recommended, although supporting evidence remains limited. Given ongoing organ shortages, it is critical to determine whether excluding donors with marginally low RLVs is justified. The objective of this study was to evaluate the safety of living liver donors with RLV < 30% after laparoscopic right hemihepatectomy.

**Methods:**

Prospectively collected data were analysed from living liver donors who underwent purely laparoscopic right hemihepatectomy between January 2022 and December 2024. The primary endpoint was posthepatectomy liver failure (PHLF), defined according to the International Study Group of Liver Surgery criteria. Secondary outcomes included the achievement of textbook outcomes in liver surgery, postoperative liver function trends, complications (Clavien–Dindo classification), and length of hospital stay. Propensity score matching (1:3) was used to compare donors with RLV < 30% and ≥ 30%.

**Results:**

Among the 374 screened donors, 28 with RLV < 30% and 77 matched controls with RLV ≥ 30% were analysed. The mean RLV values were 28.3% and 35.7%, respectively. The incidence of PHLF was numerically higher in donors with RLV < 30%, but the difference was not statistically significant (9 of 28 (32%) *versus* 13 of 77 (17%); *P* = 0.203). Similarly, textbook outcomes in liver surgery achievement rates did not differ significantly between the two groups (24 of 28 (86%) *versus* 70 of 77 (91%); *P* = 0.430). Donors with RLV < 30% exhibited transiently higher bilirubin levels (*P* = 0.007) and earlier international normalized ratio peaks (*P* = 0.040) but values normalized during follow-up. No significant differences were found in other biochemical parameters, complications, or hospitalizations.

**Conclusions:**

In this cohort, RLV < 30% was not statistically associated with worse PHLF or composite perioperative safety outcomes. Although PHLF occurred more frequently in donors with RLV < 30%, most events were grade A and postoperative biochemical dysfunction resolved during follow-up. These findings suggest that, in carefully selected donors undergoing minimally invasive donor hepatectomy at experienced centres, an RLV < 30% may not necessarily represent a strict binary threshold, although caution is warranted given the limited sample size.

## Teaser

This retrospective single-center study evaluated outcomes of living liver donors with remnant liver volume (RLV) < 30% undergoing pure laparoscopic right hepatectomy. After propensity score matching, no statistically significant differences were observed in textbook outcomes in liver surgery or posthepatectomy liver failure compared with donors with RLV ≥ 30%, although transient elevations in bilirubin and international normalized ratio were noted in the lower RLV group and resolved during follow-up. Given the limited sample size and modest discriminatory performance of RLV alone, these findings suggest that an RLV slightly below 30% was not clearly associated with worse short-term outcomes in this selected cohort, and should be interpreted cautiously pending further validation.

## Introduction

Living-donor liver transplantation (LDLT) has become a critical option for patients with end-stage liver disease and hepatocellular carcinoma, particularly in regions with a shortage of deceased donor organs^1–3^. Donor safety is the cornerstone of LDLT, as donors accept surgical risk without direct medical benefit. The most serious concern following donor hepatectomy is posthepatectomy liver failure (PHLF), which is closely linked to the size and function of the liver remnant^4–6^.

To safeguard donors, the current expert consensus recommends preserving a remnant liver volume (RLV) of at least 30%^7^. This threshold, however, is largely derived from expert consensus and observational data rather than robust prospective evidence^7^. Whereas some studies^8,9^ have suggested that donor outcomes with RLV < 30% may still be acceptable, others have reported an increased risk of morbidity and liver failure^10^. Previous research is further limited by heterogeneity in surgical approaches (open *versus* laparoscopic techniques), very small numbers of donors with RLV < 30%, lack of adjustment for baseline imbalances, and a focus restricted to in-hospital outcomes without characterization of longitudinal recovery.

In the current era of minimally invasive donor surgery, pure laparoscopic donor hepatectomy has been associated with reduced perioperative morbidity compared with open approaches, but evidence is scarce on whether it mitigates the risks in donors with borderline RLV. Moreover, no previous study has evaluated donor safety using Textbook Outcome in Liver Surgery (TOLS), a recently developed composite benchmark capturing multiple dimensions of perioperative success. Similarly, dynamic analyses of postoperative biochemical trajectories have rarely been applied to this context.

Therefore, this study aimed to investigate whether living liver donors with RLV < 30% undergoing pure laparoscopic right hepatectomy experience clearly excess risk compared with those with RLV ≥ 30%. By employing propensity score matching (PSM), PHLF as the primary endpoint, along with TOLS and longitudinal biochemical kinetics as complementary outcomes, this study seeks to provide refined evidence to inform donor selection policies in the context of minimally invasive donor hepatectomy.

## Methods

### Study design and population

This single-centre observational cohort study evaluated the clinical impact of RLV in living liver donors who underwent right hepatectomy at Samsung Medical Centre (Seoul, Korea) between 1 January 2022 and 31 December 2024, using prospectively collected clinical and laboratory data based on a standardized institutional follow-up protocol. During the study period, all living-donor right hepatectomies at the authors’ institution were performed using a pure laparoscopic approach. All operations were performed by three hepatobiliary surgeons with established experience in minimally invasive donor hepatectomy, and the study protocol was approved by the Institutional Review Board of Samsung Medical Centre (IRB No. 2025-04-083). Donors aged ≥ 18 years who underwent right hemihepatectomy or extended right hemihepatectomy using a pure laparoscopic approach were eligible; non-right-lobe resections, cases with missing preoperative volumetry, and those lacking postoperative outpatient follow-up with longitudinal laboratory measurements were excluded.

### Data collection and variables

Preoperative donor data included demographic and clinical characteristics such as sex, age, body mass index (BMI), operative date, presence of hypertension or diabetes mellitus, and calculated remnant liver percentage. The primary exposure was preoperative RLV (%), measured using contrast-enhanced donor computed tomography (CT) angiography with volumetric analysis primarily based on portal venous phase images. Three-dimensional liver segmentation and volumetric assessment were performed using a deep learning–based platform previously validated by the authors’ group^11^. Automated segmentation was manually refined when necessary. The planned transection plane and volumetric results were independently reviewed before surgery by a transplant surgery fellow and subsequently confirmed by the operating surgeons before surgery. Total liver volume, estimated graft volume, and RLV were calculated according to the predefined transection plane. Additional perioperative and pathological data were collected, including the degree of macrovesicular and microvesicular steatosis on histological evaluation of intraoperative liver specimens, total operative time, and cumulative Pringle manoeuvre duration. Preoperative laboratory parameters were also recorded, including the most recent serum levels of albumin, aspartate aminotransferase (AST), alanine aminotransferase (ALT), total bilirubin, alkaline phosphatase (ALP), platelet count, and international normalized ratio (INR).

### Outcome measures

The primary outcome was PHLF defined and graded according to the International Study Group of Liver Surgery criteria^12^. Secondary outcomes included achievement of TOLS, overall and major postoperative complications (classified by the Clavien–Dindo system), length of hospital stay, unplanned readmission, and trajectories of postoperative laboratory parameters (total bilirubin, INR, AST, ALT, ALP, and platelet count). TOLS was defined according to the international Delphi consensus as the absence of mortality, major complications [Clavien–Dindo grade ≥ III], reoperation, readmission, bile leak, and PHLF^13^. Laboratory values were obtained before surgery; on postoperative days (POD) 1, 2, 3, and 5 during hospitalization; and at the first and second outpatient visits. In addition, the optimal cut-off value of the RLV for achieving favourable outcomes was evaluated.

### Postoperative liver function analysis

To analyse systematically the kinetics of liver function recovery, four metrics were derived from these serial measurements (*Fig. S1*). The peak postoperative value was defined as a local maximum within the predefined observation window. In plateau patterns or gradual increases within the normal range, a distinct peak could not be identified. Delta X (ΔX) was defined as the time interval from the preoperative measurement to the peak postoperative value. Delta Y (ΔY) represents the absolute difference between the preoperative baseline and the postoperative peak value. Delta Z (ΔZ) was defined as the time interval from the peak postoperative value to the final measurement at the second outpatient visit. Additionally, two rate-based metrics were calculated: ΔY/ΔX, reflecting the velocity of increase from the baseline to the peak, and Delta Y/Delta Z (ΔY/ΔZ), indicating the rate of recovery from the peak value to the final measurement at the second outpatient visits. For postoperative laboratory parameters, missing values at individual time points, due to incomplete follow-up, were handled using a complete-case approach; kinetic parameters were calculated only when sufficient longitudinal measurements were available for peak detection.

### Statistical analysis

Baseline characteristics were presented as mean(standard deviation (s.d.)) or median (interquartile range (i.q.r.)) as appropriate based on the normality assumption for continuous variables; and as frequencies with percentages for categorical variables. Group comparisons were assessed using Student’s *t* test or the Mann–Whitney *U* test as appropriate for continuous variables, and the χ^2^ test or Fisher’s exact test for categorical variables.

To minimize selection bias between donors with < 30% and ≥ 30% RLV, PSM was performed using a 1:3 nearest-neighbour algorithm without replacement. Patients were matched based on the following variables: age, sex, BMI, degree of macrovesicular and microvesicular steatosis, operative time, total Pringle time, and preoperative liver function markers (AST, ALT, albumin, total bilirubin, ALP, platelet count, and INR). Operative time and total Pringle time were included as intraoperative surrogate measures for procedural burden and ischaemic exposure, respectively, because both may influence postoperative liver function recovery after donor hepatectomy^14,15^. The balances in distributions of variables were checked using the absolute value of the standardized mean difference (ASMD). An acceptable balance was declared as ASMD less than 0.2.

For the matched cohort, generalized estimating equations were used to estimate beta coefficients (β) and 95% confidence intervals (c.i.) for continuous outcomes; and to estimate odds ratios (ORs) and 95% c.i. for binary outcomes. To identify the optimal cut-off value of RLV associated with favourable outcomes, receiver operating characteristic (ROC) curve analysis with area under the curve (AUC) was performed. A two-sided *P*-value < 0.05 was considered statistically significant. All statistical analyses were performed using R software (version 4.5.1; R Foundation for Statistical Computing, Vienna, Austria).

## Results

### Donor characteristics

In the unmatched cohort (374), the median age was 37 years, 186 (49.7%) were men, and the median BMI was 24.64 kg/m^2^. The median operative time and total Pringle time were 188 and 18 minutes (min), respectively. Before matching, notable imbalances were observed in BMI, hepatic steatosis, operative time, diabetes mellitus, AST, platelet count, and INR; the median RLV was 28.55% in the < 30% group and 34.97% in the ≥ 30% group (*Table S1*).

After applying 1:3 PSM, 105 donors were included (28 (26.7%) with RLV < 30% and 77 (73.3%) with RLV ≥ 30%). After matching, the ASMD values for all variables were reduced below 0.20, indicating an improved covariable balance between the groups. In the matched cohort (105), the median age was 40 years, 51 (49%) were men, and the median BMI was 24.0 kg/m^2^. The median operative and total Pringle times were 185 and 17 min, respectively. The baseline laboratory profiles were comparable between the groups, indicating no significant differences after matching. The median RLV was 28.55% (i.q.r. 27.26–29.43%; range, 26.0–29.94%) in the < 30% group and 34.85% (i.q.r. 33.04–38.00%; range, 30.0–44.6%) in the ≥ 30% group (*Table 1*).

**Table 1:** Baseline characteristics of living liver donors after PSM by RLV

	After PSM (1:3)				
	Total (*n* = 105)	RLV < 30% (*n* = 28)	RLV ≥ 30% (*n* = 77)	ASMD	P*
Age (years), median	39	41.5	39	0.1413	0.500
**Sex**					
Male	51 (48.6%)	14 (50%)	37 (48%)	0.0390	0.858
Female	54 (51.4%)	14 (50%)	40 (52%)		
Body mass index, median	24.12	24.30	23.75	0.0917	0.662
**Macrovesicular steatosis**					
Grade 1: none–mild (< 5%)	46 (43.8%)	13 (46%)	33 (43%)	0.1235	0.851
Grade 2: mild (5–10%)	53 (50.5%)	13 (46%)	40 (52%)		
Grade 3: moderate (10–30%)	6 (5.7%)	2 (7%)	4 (5%)		
Grade 4: severe (> 30%)	0 (0.0%)	0 (0%)	0 (0%)		
**Microvesicular steatosis**					
Grade 1: none–mild (< 5%)	47 (44.8%)	13 (46%)	34 (44%)	0.1446	0.821
Grade 2: mild (5–10%)	43 (41.0%)	12 (43%)	31 (40%)		
Grade 3: moderate (10–30%)	15 (14.3%)	3 (11%)	12 (16%)		
Grade 4: severe (> 30%)	0 (0.0%)	0 (0%)	0 (0%)		
Duration of operation (min), median	188.00	176.50	193.00	0.1837	0.440
Total Pringle time (min), median	18.00	15.50	18.00	0.1530	0.535
RLV (%), median	33.60	28.55	34.85	2.7271	< 0.001
**Diabetes mellitus**					
No	104 (99.0%)	28 (100%)	76 (99%)	0.1622	1.000
Yes	1 (1.0%)	0 (0%)	1 (1%)		
**Hypertension**					
No	98 (93.3%)	27 (96%)	71 (92%)	0.1831	0.671
Yes	7 (6.7%)	1 (4%)	6 (8%)		
Albumin (g/dl), mean	4.35	4.37	4.35	0.0720	0.739
AST (U/l), mean	17.60	17.36	17.69	0.0805	0.695
ALT (U/l), mean	18.16	17.75	18.31	0.0564	0.792
Total bilirubin (mg/dl), mean	0.57	0.59	0.57	0.0816	0.716
Platelet count (×10^9^/l), mean	232.36	231.79	232.57	0.0177	0.939
INR, mean	1.01	1.01	1.00	0.1379	0.513

Values are *n* (%) unless otherwise stated. All covariates achieved acceptable balance after PSM (ASMD < 0.2) and complete for all donors, with no missing values. PSM, propensity score matching; RLV, remnant liver volume; ASMD, absolute standardized mean difference; min, minute; AST, aspartate aminotransferase; ALT, alanine aminotransferase; INR, international normalized ratio. *P values were obtained using generalized estimating equations in the matched cohort.

### PHLF

Among the 105 matched donors, 22 (21.0%) developed any-grade PHLF, including 14 cases of grade A (13.3%) and 8 cases of grade B (7.6%); no grade C PHLF occurred. PHLF occurred more frequently in donors with RLV < 30% than in those with RLV ≥ 30% (9 of 28 (32.1%) *versus* 13 of 77 (16.9%)); however, there was no significant association between lower RLV and the incidence of PHLF (OR 1.94, 95% c.i. 0.70 to 5.37; *P* = 0.203) (*Table 2*). The mean(s.d.) RLV was lower in donors with PHLF than in those without PHLF, although this difference was not statistically significant (32.2(4.8)% *versus* 33.8(4.2)%; *P* = 0.141).

**Table 2:** Posthepatectomy liver failure and TOLS according to RLV

	Total (*n* = 105)	Group RLV < 30% (*n* = 28)	Group RLV ≥ 30% (*n* = 77)	Odds ratio*	*P*†
**PHLF**	22 (21.0%)	9 (32%)	13 (17%)	1.938 (0.70, 5.37)	0.203
Grade A	14 (13.3%)	6 (21%)	8 (10%)		
Grade B	8 (7.6%)	3 (11%)	5 (7%)		
Grade C	0 (0.0%)	0 (0%)	0 (0%)		
TOLS	94 (89.5%)	24 (86%)	70 (91%)	0.588 (0.16, 2.20)	0.430
**TOLS failure component**					
CD ≥ III‡	5 (4.8%)	1 (3.6%)	4 (5.2%)		
Readmission§	4 (3.8%)	1 (4%)	3 (4%)		
Bile leakage¶	1 (1.0%)	0 (0%)	1 (1%)		
Grade B/C PHLF#	8 (7.6%)	3 (11%)	5 (7%)		
Mortality**	0 (0.0%)	0 (0%)	0 (0%)		

Values are *n* (%) unless otherwise stated. *Values in parentheses are 95% confidence intervals. Grading of PHLF according to International Study Group of Liver Surgery criteria. †*P* values for PHLF and TOLS were obtained using generalized estimating equations for binary outcomes in the matched cohort. ‡90-day postoperative complications Clavien–Dindo grade III or higher. §90-day readmission due to surgery-related complications Clavien–Dindo grade III or higher. ¶Postoperative bile leakage of grades B and C. #Posthepatectomy liver failure of grades B and C. **In-hospital and 90-day mortality. TOLS, Textbook Outcome in Liver Surgery; RLV, remnant liver volume; PHLF, posthepatectomy liver failure.

### TOLS

Among the 105 matched donors, 94 of 105 (89.5%) achieved TOLS. The rate was slightly lower in donors with RLV < 30% than in those with RLV ≥ 30% (24 of 28 (85.7%) *versus* 70 of 77 (90.9%)), although this difference was not statistically significant (OR 0.59, 95% c.i. 0.16 to 2.20; *P* = 0.430) (*Table 2*). The individual TOLS components are summarized in *Table 2*.

### Postoperative complications

Postoperative complications were uncommon in both groups. After PSM, complications occurred in 2 of 28 donors (7.1%) with RLV < 30% and 7 of 77 donors (9.1%) with RLV ≥ 30%. There were no significant differences in major complications (1 of 28 (3.6%) *versus* 4 of 77 (5.2%); *P* = 0.860) or any-grade complications (2 of 28 (7.1%) *versus* 7 of 77 (9.1%); *P* = 0.840). Detailed complication profiles before and after matching are provided in *Table S2*.

### Hospitalization

In the matched cohort, the mean length of hospital stay was comparable between groups, with no significant association according to RLV (7.93 *versus* 7.44 days; β = 0.57, 95% c.i. −0.24 to 1.37; *P* = 0.170). Ninety-day readmission occurred in 1 of 28 donors (3.6%) in the < 30% group and 3 of 77 donors (3.9%) in the ≥ 30% group, showing no significant difference (OR 1.00, 95% c.i. 0.10 to 10.04; *P* = 1.000).

### Postoperative laboratory test trends over time

Median values of laboratory parameters were analysed from preoperative to postoperative days (POD1, POD2, POD3, POD5) and outpatient follow-up (first outpatient visit (mean POD26), second outpatient visit (mean POD168)) for donors with RLV < 30% (28) *versus* RLV ≥ 30% (77) (*Table S3*).

Total bilirubin showed consistently higher median values in the < 30% group from POD1 to POD5 compared with the ≥ 30% group, with peak median values at POD2 (3.75 *versus* 2.40 mg/dl). By the second outpatient visit, the median bilirubin values normalized and converged between groups. INR also peaked early after surgery (POD2) with slightly higher median levels in the < 30% group, before gradually declining to preoperative values at subsequent outpatient visits. The overall pattern and normalization rates were similar between the groups. AST, ALT, and platelet count showed broadly similar postoperative trajectories between groups. ALP displayed an initial postoperative decrease, followed by a gradual increase, reaching peak values at the first outpatient visit. Median ALP values were slightly higher in the < 30% group during follow-up visits (*Fig. 1*).

**Figure 1:**
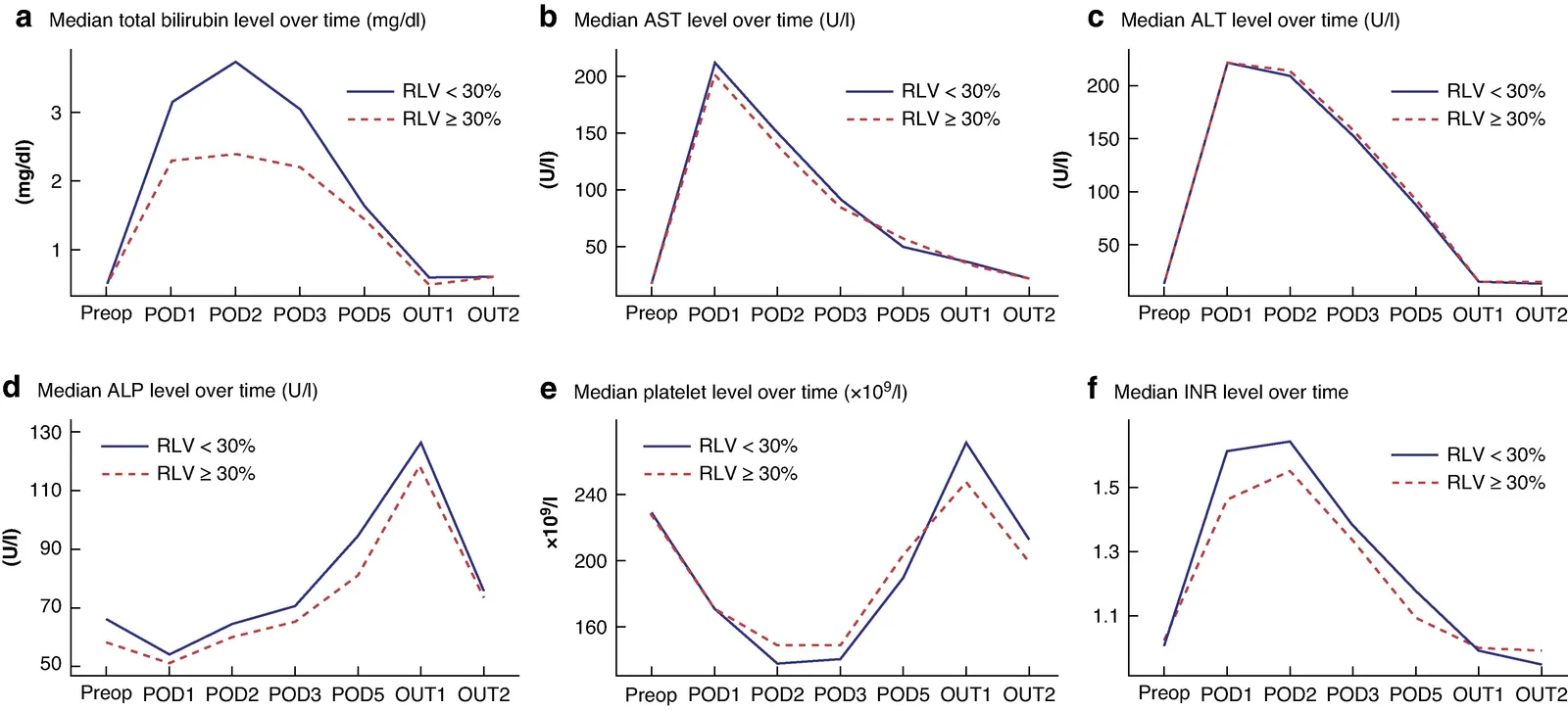
Serial postoperative laboratory trends according to RLV Median levels over time: **a** total bilirubin (mg/dl), **b** AST (U/l), **c** ALT (U/l), **d** ALP (U/l), **e** platelet ((×10^9^/l), **f** INR. Median laboratory values are shown from the preoperative period to the second outpatient visit in donors with RLV < 30% and RLV ≥ 30%. RLV, remnant liver volume; AST, aspartate aminotransferase; ALT, alanine aminotransferase; ALP, alkaline phosphatase; INR, international normalized ratio; Preop, preoperative, POD, postoperative day.

### Donor postoperative laboratory tests

*Table S4* summarizes the association between postoperative liver function dynamics and the < 30% and ≥ 30% RLV groups in the matched cohort (105). Postoperative liver function dynamics were analysed using the following kinetic parameters: time to peak (ΔX), peak level from baseline (ΔY), velocity to peak from baseline (ΔY/ΔX), and velocity from peak to end (ΔY/ΔZ). *Figure 2* illustrates these differences with β estimates and 95% c.i., highlighting both the magnitude and direction of group differences.

**Figure 2:**
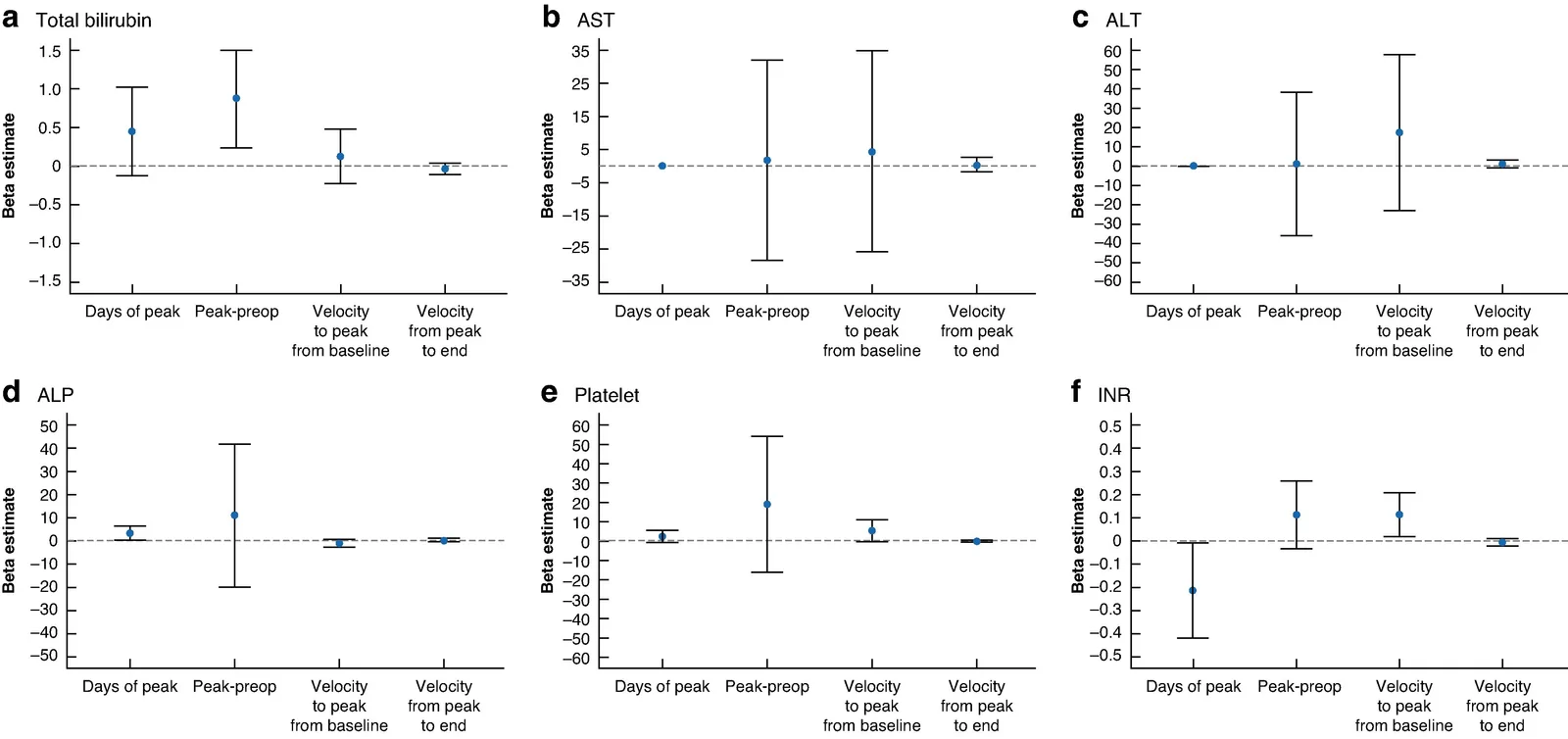
Postoperative liver function kinetics according to RLV **a** Total bilirubin, **b** AST, **c** ALT, **d** ALP), **e** platelet, **f** INR. Effect estimates and 95% confidence intervals are shown for kinetic parameters in donors with RLV < 30% compared with those with RLV ≥ 30%. RLV, remnant liver volume; AST, aspartate aminotransferase; ALT, alanine aminotransferase; ALP, alkaline phosphatase; INR, international normalized ratio; Preop, preoperative.

For total bilirubin, the RLV < 30% group showed significantly higher peak elevation from baseline than the RLV ≥ 30% group (ΔY: 3.40 *versus* 2.58 mg/dl; β = 0.87, 95% c.i. 0.237 to 1.499; *P* = 0.007), whereas no significant associations were observed for ΔX, ΔY/ΔX, or ΔY/ΔZ.

For ALT, the RLV < 30% group reached peak ALT significantly earlier than the RLV ≥ 30% group (ΔX: 1.11 *versus* 1.26 days; β = −0.16, 95% c.i. −0.320 to −0.008; *P* = 0.039), whereas ΔY, ΔY/ΔX, and ΔY/ΔZ showed no significant differences.

Regarding ALP, the RLV < 30% group demonstrated a significantly delayed peak compared with the RLV ≥ 30% group (ΔX: 22.11 *versus* 18.79 days; β = 3.17, 95% c.i. 0.108 to 6.224; *P* = 0.042). No group differences were observed for ΔY, ΔY/ΔX, or ΔY/ΔZ.

For INR, the RLV < 30% group reached peak values earlier (ΔX: 1.62 *versus* 1.82 days; β = −0.22, 95% c.i. −0.422 to −0.009; *P* = 0.040) and exhibited a faster rate of increase (ΔY/ΔX: 0.49/day *versus* 0.38/day; β = 0.11, 95% c.i. 0.017 to 0.205; *P* = 0.021). In contrast, AST and platelet count showed no significant between-group differences across the kinetic parameters. Detailed kinetic results are provided in *Table S4* and *Fig. 2*.

### Exploratory analysis of RLV in relation to adverse outcomes

ROC curve analysis was performed to examine whether RLV showed a potential inflection point in relation to composite adverse outcomes (TOLS failure or PHLF). The AUC was 0.592, and the optimal cut-off determined by Youden’s index was 28.37%, with a sensitivity of 0.28 and a specificity of 0.93. When donors were stratified by RLV into three groups (< 28.4%, 28.4–30%, and ≥ 30%), the incidence of composite failure was 53.8%, 20.0%, and 19.4%, respectively. Donors with RLV < 28.4% showed a significantly higher rate of composite failure than those with RLV ≥ 28.4% (Fisher’s exact *P* = 0.014, OR 4.82) (*Fig. 3*).

**Figure 3:**
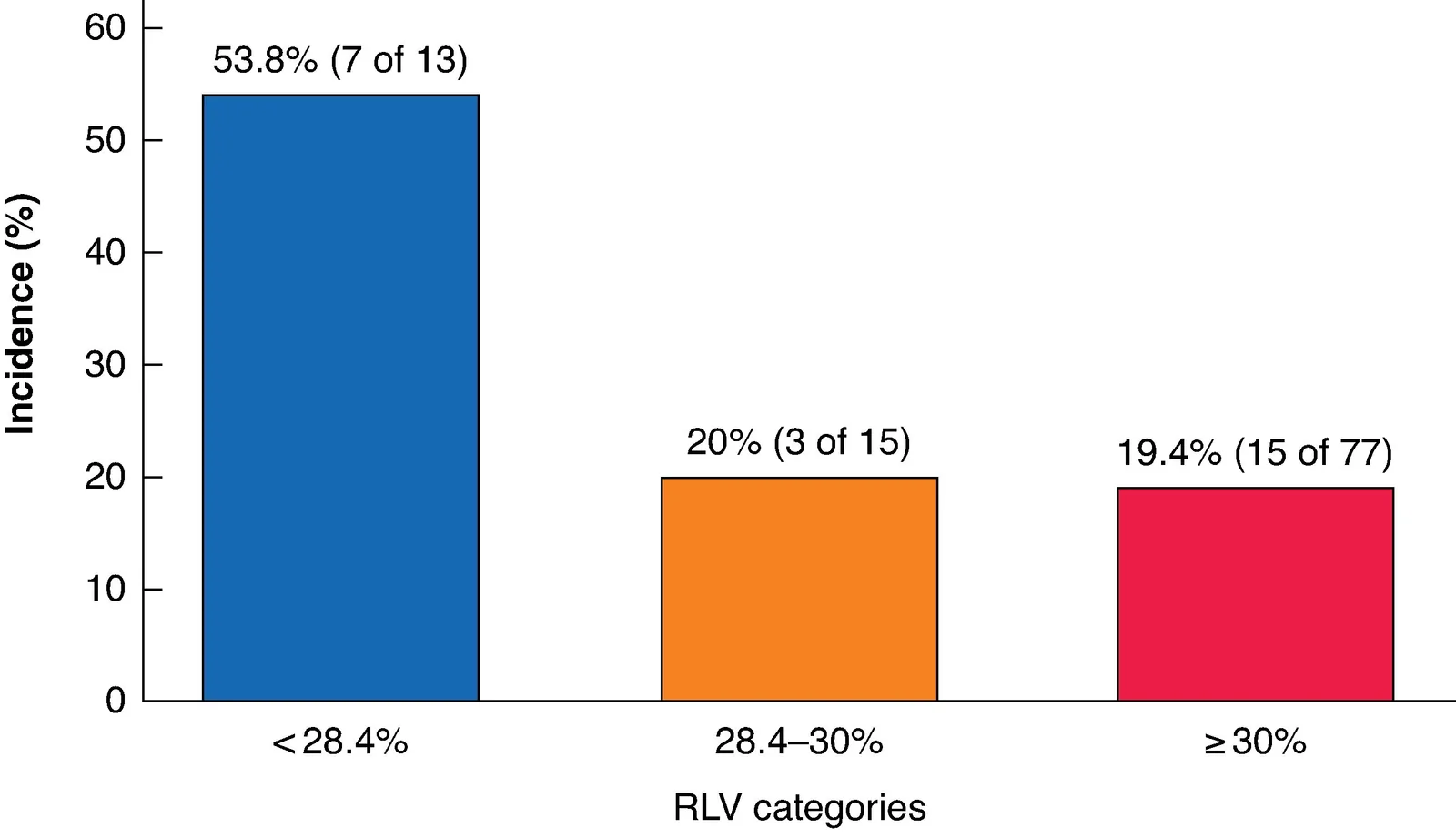
Incidence of composite adverse outcomes according to RLV strata Composite adverse outcomes were defined as TOLS failure or PHLF rate by RLV. Donors were stratified into three groups based on exploratory ROC-derived RLV categories: < 28.4%, 28.4–30%, and ≥ 30%. Fisher’s exact *P* = 0.014; OR 4.82. RLV, remnant liver volume; TOLS, Textbook Outcome in Liver Surgery; PHLF, posthepatectomy liver failure, ROC, receiver operating characteristic; OR, odds ratio.

## Discussion

In this single-centre cohort of living liver donors undergoing pure laparoscopic right hepatectomy, no statistically significant differences in PHLF or TOLS were observed between donors with RLV < 30% and those with RLV ≥ 30%. Early postoperative total bilirubin and INR were transiently higher in the < 30% group but normalized during follow-up. Other laboratory parameters (AST, ALT, and platelet count) were comparable, and ALP showed a delayed peak at the first outpatient visit. Collectively, these data suggest that, within carefully selected donors in experienced minimally invasive liver surgery (MILS) settings, a conventional 30% RLV threshold may warrant re-evaluation rather than being considered an absolute contraindication.

Evidence regarding donor safety below the conventional RLV ≥ 30% threshold is mixed: some studies suggest that RLV < 30% can be safe, whereas others indicate a higher risk^8–10^. This controversy likely reflects mixed surgical approaches (open and laparoscopic), very small < 30% subgroups with limited statistical power, and operator-dependent volumetry with non-standardized definitions that introduce measurement variability. Most previous studies also confined outcomes to the in-hospital period and did not model serial laboratory trajectories into outpatient follow-up. To address these gaps, the authors analysed a homogeneous pure laparoscopic donor cohort, applied PSM on key donor characteristics, and evaluated predefined clinical endpoints alongside repeated measures analyses of laboratory data extending into the second outpatient visit.

PHLF was used as the primary endpoint because it reflects early postoperative liver function and functional reserve after hepatectomy^12,16^. TOLS, although originally developed as a benchmark in liver surgery, was assessed as a secondary endpoint because its composite structure provides a stringent measure of overall perioperative donor safety^13,17^. This endpoint hierarchy enabled evaluation of both volume-related functional recovery and the broader surgical safety profile within the same matched cohort.

In this cohort, no statistically significant differences in PHLF or TOLS were observed between donors with RLV < 30% and those with RLV ≥ 30%, although a numerically higher rate of any-grade PHLF was observed in the RLV < 30% group. Owing to the small number of donors with RLV < 30%, the confidence intervals were wide, and clinically meaningful differences cannot be excluded. The absence of statistical significance should therefore not be interpreted as evidence of no association between low RLV and PHLF. Nevertheless, most PHLF events in the RLV < 30% group were grade A, which is generally considered a biochemical alteration without clinical consequences and is typically managed with observation alone, and the associated postoperative biochemical changes were transient.

In the repeated measures model, the group and group-by-time interaction terms were significant for INR and total bilirubin only, whereas AST, ALT, and platelet counts were comparable between RLV < 30% and RLV ≥ 30% donors, indicating that the extent of acute hepatocellular injury was similar, whereas synthetic and excretory reserves were more sensitive to smaller remnant volume^18^. ALP demonstrated a delayed postoperative rise with a peak during early follow-up; moreover, the time to peak was significantly longer in the < 30% group. This pattern aligns with the well described lag in biliary epithelial/canalicular remodelling after hepatectomy, during which ALP may remain elevated despite recovery of bilirubin and INR^19,20^. Importantly, the direction of change was towards recovery in both groups, and all laboratory values normalized on follow-up, consistent with physiological regeneration rather than persistent cholestasis.

At the authors’ centre, graft selection is guided not only by donor RLV but also by the need to ensure an adequate graft-to-recipient weight ratio. In selected cases, larger grafts were required to provide sufficient graft volume for recipients with advanced liver disease, particularly when no alternative suitable donors were available. However, acceptance of donors with borderline RLV was based on an integrated assessment of donor liver quality, baseline liver function, operative feasibility, and overall donor safety, rather than on recipient requirements alone.

Exploratory ROC analysis suggested a potential inflection point around 28.4% for composite adverse outcomes. However, the discriminatory performance was modest (AUC = 0.592), indicating that RLV alone had limited ability to distinguish between donors with and without adverse outcomes. This threshold should be interpreted cautiously and regarded as hypothesis-generating rather than clinically actionable. These findings support the need for individualized risk assessment in donors with borderline RLV, whereas further validation in larger, multicentre cohorts is required before any modification of current donor selection policies can be considered.

The results of this study should be interpreted cautiously for several reasons. First, this was a retrospective, single-centre analysis, which entails inherent risks of selection and information bias despite the use of PSM to address baseline imbalances. Although PSM was performed to reduce measured confounding, this approach cannot account for unmeasured variables. In addition, inclusion of intraoperative variables (operative time and total Pringle time) may have introduced postexposure adjustment or overmatching. Anatomical variations in vascular or biliary structures, microarchitectural parenchymal quality beyond histologic steatosis, and intraoperative haemodynamic fluctuations may have influenced postoperative recovery. Therefore, residual confounding cannot be entirely excluded, and causal inferences should be made with caution. Second, because this study exclusively included donors undergoing pure laparoscopic right hepatectomy at a high-volume centre, the generalizability of the authors’ findings to open or hybrid approaches and to centres with differing levels of expertise may be limited. Third, the overall sample size was limited, which may have reduced statistical power and the precision of the effect estimates. Finally, RLV was estimated using preoperative CT-based volumetry, and minor discrepancies between the planned and actual transection planes may have occurred. Although postoperative CT imaging was routinely obtained, systematic volumetric reassessment was not performed. Future studies should incorporate not only standardized postoperative volumetric analysis but also functional assessment of the future liver remnant, as volume alone does not necessarily reflect functional capacity. Functional assessment (for example, indocyanine green clearance, hepatobiliary scintigraphy using Tc-mebrophenin, or functional MRI) should be carefully considered, particularly in donors with borderline RLV.

In conclusion, in a pure laparoscopic donor hepatectomy cohort, donors with RLV just below 30% did not have statistically worse PHLF or TOLS outcomes compared with those with RLV ≥ 30%. However, PHLF occurred more frequently in donors with RLV < 30%, warranting cautious interpretation. Most PHLF events were grade A, and early increases in bilirubin and INR resolved during follow-up. Taken together, these findings indicate that, within carefully selected donors in experienced MILS settings, the 30% threshold may not necessarily represent a strict binary cut-off, but rather should be interpreted as part of a broader clinical context.

## Supplementary Material

zrag116_Supplementary_Data

## Data Availability

The data supporting the findings of this study are available from the corresponding author upon reasonable request.

## References

[1] Goldaracena N, Gorgen A, Doyle A, Hansen BE, Tomiyama K, Zhang W et al Live donor liver transplantation for patients with hepatocellular carcinoma offers increased survival vs. deceased donation. J Hepatol 2019;70:666–673 10.1016/j.jhep.2018.12.029 30630009

[2] Lee S-G . Asian contribution to living donor liver transplantation. J Gastroenterol Hepatol 2006;21:572–574 10.1111/j.1440-1746.2006.04283.x 16638101

[3] Mittler J, Pascher A, Jonas S, Pratschke J, Neumann UP, Langrehr JM et al Adult living donor liver transplantation: living donation of the right liver lobe. Langenbecks Arch Surg 2007;392:657–662 10.1007/s00423-007-0187-0 17443341

[4] Feng Y, Han Z, Wang X, Chen H, Li Y. Association of graft-to-recipient weight ratio with the prognosis following liver transplantation: a meta-analysis. J Gastrointest Surg 2020;24:1869–1879 10.1007/s11605-020-04598-3 32306226

[5] Schreckenbach T, Liese J, Bechstein WO, Moench C. Posthepatectomy liver failure. Dig Surg 2012;29:79–85 10.1159/000335741 22441624

[6] Allard M-A, Adam R, Bucur P-O, Termos S, Cunha AS, Bismuth H et al Posthepatectomy portal vein pressure predicts liver failure and mortality after major liver resection on noncirrhotic liver. Ann Surg 2013;258:822–829; discussion 829–830 10.1097/SLA.0b013e3182a64b38 24045452

[7] Soin AS, Chaudhary RJ, Pahari H, Pomfret EA. A worldwide survey of live liver donor selection policies at 24 centers with a combined experience of 19 009 adult living donor liver transplants. Transplantation 2019;103:e39–e47 10.1097/TP.0000000000002475 30308575

[8] Zuckerman J, Gorgen A, Acuna SA, Abreu P, Goldaracena N, Galvin Z et al Outcomes of highly selected live donors with a future liver remnant less than or equal to 30%: a matched cohort study. Transplantation 2021;105:2397–2403 10.1097/TP.0000000000003559 33239541

[9] Kim SH, Kim YK, Lee SD, Park S-J. Selection and outcomes of living donors with a remnant volume less than 30% after right hepatectomy. Liver Transpl 2013;19:872–878 10.1002/lt.23677 23695974

[10] Kim S-H, Kim K-H, Cho H-D. Donor safety of remnant liver volumes of less than 30% in living donor liver transplantation: a systematic review and meta-analysis. Clin Transplant 2023;37:e15080 10.1111/ctr.15080 37529969

[11] Oh N, Kim J-H, Rhu J, Jeong WK, Choi G-S, Kim J et al Comprehensive deep learning-based assessment of living liver donor CT angiography: from vascular segmentation to volumetric analysis. Int J Surg 2024;110:6551–6557 10.1097/JS9.0000000000001829 PMC11487025 38869975

[12] Rahbari NN, Garden OJ, Padbury R, Brooke-Smith M, Crawford M, Adam R et al Posthepatectomy liver failure: a definition and grading by the International Study Group of Liver Surgery (ISGLS). Surgery 2011;149:713–724 10.1016/j.surg.2010.10.001 21236455

[13] Gorgec B, Benedetti Cacciaguerra A, Pawlik TM, Aldrighetti LA, Alseidi AA, Cillo U et al An international expert Delphi consensus on defining Textbook Outcome in Liver Surgery (TOLS). Ann Surg 2023;277:821–828 10.1097/SLA.0000000000005668 PMC10082050 35946822

[14] Morino K, Seo S, Yoh T, Toda R, Yoshino K, Nishio T et al Impact of the intermittent Pringle maneuver for predicting post-hepatectomy liver failure: a cohort study of 597 consecutive patients. World J Surg 2023;47:1058–1067 10.1007/s00268-023-06904-x 36633645

[15] Wei X, Zheng W, Yang Z, Liu H, Tang T, Li X et al Effect of the intermittent Pringle maneuver on liver damage after hepatectomy: a retrospective cohort study. World J Surg Oncol 2019;17:142 10.1186/s12957-019-1680-y PMC6693131 31409370

[16] Vitello DJ, Shah D, Ko B, Brajcich BC, Peters XD, Merkow RP et al Establishing the clinical relevance of grade A post-hepatectomy liver failure. J Surg Oncol 2024;129:745–753 10.1002/jso.27570 PMC10922784 38225867

[17] Bhatti ABH, Naqvi W, Ali N, Khan NY, Zia HH, Faiz BY et al Textbook outcome among voluntary donors undergoing major living donor hepatectomy. Langenbecks Arch Surg 2022;407:2905–2913 10.1007/s00423-022-02578-6 PMC9171097 35670859

[18] de Graaf W, Heger M, Spruijt O, Maas A, de Bruin K, Hoekstra R et al Quantitative assessment of liver function after ischemia-reperfusion injury and partial hepatectomy in rats. J Surg Res 2012;172:85–94 10.1016/j.jss.2010.06.038 20869070

[19] Muraoka I, Soyama A, Takatsuki M, Tomonaga T, Hidaka M, Kanematsu T et al Transition of serum alkaline phosphatase isoenzymes during liver regeneration in humans. Hepatogastroenterology 2013;60:1476–1478 10.5754/hge11097 23933940

[20] Kaneko K, Kamimoto K, Miyajima A, Itoh T. Adaptive remodeling of the biliary architecture underlies liver homeostasis. Hepatology 2015;61:2056–2066 10.1002/hep.27685 25572923

