# Supplementary material for: Safety of living liver donors with remnant liver volume below 30% after laparoscopic living donor hepatectomy
Source: BJS Open. 2026 Nov 13;10(6):zrag116. doi: 10.1093/bjsopen/zrag116 (PMC13760211; doi:10.1093/bjsopen/zrag116)
Supplement: Supplementary file 1 [file zrag116_supplementary_data.docx]

**Is Remnant Liver Volume <30% an Absolute Contraindication in Purely Laparoscopic Living Donor Hepatectomy?**

**Running head**

Donor Safety below 30% Remnant Liver Volume

Sukmin Gwon^1,*^, Namkee Oh^1,*^, Sang Ah Chi^2^, Sunghae Park^1^, Eunjin Lee^1^, Youngju Ryu^1^, Jiyoung Baik^1^, Hayeon Do^1^, Jaehyun Kwon^1^, Hansang Park^1^, Inho Lee^1^, Yoonsun Choi^1^, Jinsoo Rhu^1^, Kyunga Kim^2^, Jongman Kim^1^, Gyu-Seong Choi^1†^

^1^Department of Surgery, Samsung Medical Center, Sungkyunkwan University School of Medicine, Seoul, Republic of Korea

^2^ Biomedical Statistics Center, Research Institute for Future Medicine, Samsung Medical Center, Seoul, Republic of Korea

*These authors contributed equally as first authors

^†^**Corresponding Author:**

Gyu-Seong Choi, MD, PhD

Department of Surgery, Samsung Medical Center, Sungkyunkwan University School of Medicine,

81 Irwon-ro, Gangnam-gu, Seoul 06351, Republic of Korea

med9370@gmail.com

**Supplementary Materials – Index**

| **Supplementary Figures and Tables** |  |
| --- | --- |
| Figure S1 | *pag. 3* |
| Table S1 | *pag. 4* |
| Table S2 | *pag. 5* |
| Table S3 | *pag. 6* |
| Table S4 | *pag. 7* |
|  |  |
|  |  |

**Supplementary Figures and Tables**

**figure S1.** Kinetic Parameters used to assess postoperative liver function dynamics.

(ΔX indicates the time from baseline to peak, ΔY indicates the magnitude of change from baseline to peak, and ΔZ indicates the time from peak to the final follow-up measurement. ΔY/ΔX represents the rate of increase to peak, and ΔY/ΔZ represents the rate of recovery after peak.)

**Supplementary Table S1.**  Baseline Characteristics of Living Liver Donors Before Propensity Score Matching by Remnant Liver Volume

|  |  | **Before PSM** | | | | |
| --- | --- | --- | --- | --- | --- | --- |
|  |  | **Total** | **RLV <30%** | **RLV ≥30%** | **ASMD** | ***P* value** |
|  |  | 374 | 30 (8.0%) | 344 (92.0%) | — | — |
| Age | Median | 37 | 39.5 | 37 | 0.1185 | 0.3491 |
| Sex | Male, n (%) | 186 (49.7%) | 16 (53.3%) | 170 (49.4%) | 0.0784 | 0.8252 |
|  | Female, n (%) | 188 (50.3%) | 14 (46.7%) | 174 (50.6%) |  |  |
| Body mass index | Median | 24.64 | 24.30 | 24.69 | 0.2023 | 0.4505 |
| Macrovesicular steatosis | Grade 1: None–mild (<5%) | 131 (35.0%) | 15 (50.0%) | 116 (33.7%) | 0.3579 | 0.2880 |
|  | Grade 2: Mild (5–10%) | 198 (52.9%) | 13 (43.3%) | 185 (53.8%) |  |  |
|  | Grade 3: Moderate (10–30%) | 44 (11.8%) | 2 (6.7%) | 42 (12.2%) |  |  |
|  | Grade 4: Severe (>30%) | 1 (0.3%) | 0 (0.0%) | 1 (0.3%) |  |  |
| Microvesicular steatosis | Grade 1: None–mild (<5%) | 140 (37.4%) | 15 (50.0%) | 125 (36.3%) | 0.2897 | 0.2792 |
|  | Grade 2: Mild (5–10%) | 196 (52.4%) | 12 (40.0%) | 184 (53.5%) |  |  |
|  | Grade 3: Moderate (10–30%) | 38 (10.2%) | 3 (10.0%) | 35 (10.2%) |  |  |
|  | Grade 4: Severe (>30%) | 0 (0.0%) | 0 (0.0%) | 0 (0.0%) |  |  |
| Operation time (min) | Median | 188.00 | 176.50 | 189.00 | 0.2080 | 0.1365 |
| Total Pringle time (min) | Median | 18.00 | 16.00 | 18.00 | 0.0784 | 0.7290 |
| Remnant liver volume (%) | Median | 34.50 | 28.55 | 34.97 | 2.6559 | <0.0001 |
| Diabetes mellitus | No, n (%) | 366 (97.9%) | 30 (100.0%) | 336 (97.7%) | 0.2182 | 1.0000 |
|  | Yes, n (%) | 8 (2.1%) | 0 (0.0%) | 8 (2.3%) |  |  |
| Hypertension | No, n (%) | 348 (93.0%) | 29 (96.7%) | 319 (92.7%) | 0.1763 | 0.7091 |
|  | Yes, n (%) | 26 (7.0%) | 1 (3.3%) | 25 (7.3%) |  |  |
| Albumin (g/dL) | Mean | 4.39 | 4.37 | 4.39 | 0.0551 | 0.6602 |
| AST (U/L) | Mean | 18.42 | 17.40 | 18.51 | 0.2104 | 0.8398 |
| ALT (U/L) | Mean | 18.69 | 18.60 | 18.70 | 0.0089 | 0.8760 |
| Total bilirubin (mg/dL) | Mean | 0.59 | 0.58 | 0.59 | 0.0471 | 0.5326 |
| Platelet count (×10⁹/L) | Mean | 247.89 | 229.23 | 249.51 | 0.4018 | 0.0327 |
| INR | Mean | 1.00 | 1.02 | 0.99 | 0.2940 | 0.4999 |

PSM, propensity score matching; ASMD, absolute standardized mean difference.

|  | **Before PSM** | | **After PSM** | |
| --- | --- | --- | --- | --- |
|  | **RLV ≥ 30%**  **(n = 344)** | **RLV < 30%**  **(n = 30)** | **RLV ≥ 30%**  **(n = 77)** | **RLV < 30%**  **(n = 28)** |
| Grade 1 | Surgical site infection (n = 2) | Surgical site infection (n = 1) | Surgical site infection  (n = 1) | Surgical site infection (n = 1) |
| Grade 2 | Transfusion (n = 3), Diuretics for ascites (n = 4) | None | Diuretics for ascites  (n = 2) | None |
| Grade 3a | Portal vein stenosis (n = 2), Biliary stricture (n = 5), Bile leakage (n = 7), Pleural effusion (n = 3) | Biliary stricture  (n = 1) | Bile leakage (n = 1), Pleural effusion (n = 2) | Biliary stricture  (n = 1) |
| Grade 3b | Re-operation due to bleeding  (n = 4) | None | Re-operation due to bleeding (n = 1) | None |
| Grade 4a | None | None | None | None |
| Grade 4b | None | None | None | None |
| Overall | 30/344 (8.7%) | 2/30 (6.7%) | 7/77 (9.1%) | 2/28 (7.1%) |

**Supplementary Table S2.** Postoperative Complications Before and After Propensity Score Matching According to Remnant Liver Volume

Grades according to the Clavien–Dindo classification:

**Supplementary Table S3.** Serial Trends of Postoperative Laboratory Parameters in Living Liver Donors with Remnant Liver Volume

|  |  | **Preop** | **POD1** | **POD2** | **POD3** | **POD5** | **1st outpatient visit^1^** | **2nd outpatient visit^2^** |
| --- | --- | --- | --- | --- | --- | --- | --- | --- |
| Total_  bilirubin (mg/dL) | RLV<30% | 0.50 | 3.15 | 3.75 | 3.05 | 1.65 | 0.60 | 0.60 |
|  | RLV≥30% | 0.50 | 2.30 | 2.40 | 2.20 | 1.45 | 0.50 | 0.60 |
| AST (U/L) | RLV<30% | 17.00 | 212.50 | 149.00 | 92.00 | 49.50 | 37.00 | 22.00 |
|  | RLV≥30% | 17.00 | 201.00 | 139.00 | 84.00 | 57.50 | 34.50 | 23.00 |
| ALT (U/L) | RLV<30% | 14.50 | 221.50 | 208.00 | 152.00 | 88.00 | 15.00 | 13.00 |
|  | RLV≥30% | 15.00 | 222.00 | 213.00 | 158.00 | 93.00 | 15.50 | 15.00 |
| ALP (U/L) | RLV<30% | 66.00 | 54.00 | 64.50 | 70.50 | 94.50 | 127.00 | 75.50 |
|  | RLV≥30% | 58.00 | 51.00 | 60.00 | 65.00 | 81.00 | 119.00 | 73.50 |
| Platelet (×10⁹/L) | RLV<30% | 228.50 | 171.00 | 138.00 | 141.00 | 190.00 | 272.00 | 211.50 |
|  | RLV≥30% | 227.00 | 170.00 | 149.00 | 149.00 | 203.50 | 247.50 | 199.00 |
| INR | RLV<30% | 1.01 | 1.46 | 1.55 | 1.34 | 1.10 | 1.00 | 0.99 |
|  | RLV≥30% | 0.99 | 1.61 | 1.65 | 1.39 | 1.18 | 0.99 | 0.95 |

Laboratory parameters (Preop to 2nd outpatient visit) were expressed as median values.

**Supplementary Table S4.** Postoperative Liver Function Dynamics Analyzed by Kinetic Parameters in Donors With Remnant Liver Volume

|  |  |  | **Total**  **(105)** | **Group**  **RLV<30%**  **(28)** | **Group**  **RLV≥30%**  **(77)** | **Number of**  **Obs Used (Incidence of Peak)** | **Univariable analysis** | | | |
| --- | --- | --- | --- | --- | --- | --- | --- | --- | --- | --- |
|  |  |  |  |  |  |  | **Effect size** | **95% LCL** | **95% UCL** | ***P*-value** |
| Total bilirubin | ΔX | Mean | 2.25 | 2.58 | 2.14 | 96 (91.4%) | 0.444 | -0.131 | 1.019 | 0.1303 |
|  | ΔY | Mean | 2.78 | 3.40 | 2.58 | 96 (91.4%) | 0.868 | 0.237 | 1.499 | 0.0070 |
|  | ΔY/ΔX | Mean | 1.51 | 1.58 | 1.48 | 96 (91.4%) | 0.121 | -0.230 | 0.472 | 0.4999 |
|  | ΔY/ΔZ | Mean | -0.04 | -0.07 | -0.03 | 96 (91.4%) | -0.036 | -0.107 | 0.034 | 0.3123 |
| AST | ΔX | Mean | 1.02 | 1.00 | 1.03 | 105 (100%) | -0.024 | -0.057 | 0.009 | 0.1534 |
|  | ΔY | Mean | 204.90 | 206.07 | 204.48 | 105 (100%) | 1.649 | -28.522 | 31.820 | 0.9147 |
|  | ΔY/ΔX | Mean | 202.81 | 206.07 | 201.62 | 105 (100%) | 4.268 | -26.062 | 34.597 | 0.7827 |
|  | ΔY/ΔZ | Mean | -2.88 | -2.80 | -2.91 | 105 (100%) | 0.374 | -1.848 | 2.596 | 0.7414 |
| ALT | ΔX | Mean | 1.22 | 1.11 | 1.26 | 104 (99%) | -0.164 | -0.320 | -0.008 | 0.0395 |
|  | ΔY | Mean | 225.23 | 226.43 | 224.79 | 104 (99%) | 1.230 | -35.908 | 38.367 | 0.9483 |
|  | ΔY/ΔX | Mean | 201.50 | 213.25 | 197.17 | 104 (99%) | 17.108 | -23.345 | 57.562 | 0.4071 |
|  | ΔY/ΔZ | Mean | -3.21 | -2.76 | -3.37 | 104 (99%) | 0.928 | -1.031 | 2.887 | 0.3531 |
| ALP | ΔX | Mean | 19.71 | 22.11 | 18.79 | 97 (92.4%) | 3.166 | 0.108 | 6.224 | 0.0425 |
|  | ΔY | Mean | 73.70 | 83.04 | 70.10 | 97 (92.4%) | 10.653 | -20.350 | 41.657 | 0.5006 |
|  | ΔY/ΔX | Mean | 4.75 | 3.84 | 5.10 | 97 (92.4%) | -1.280 | -2.879 | 0.319 | 0.1167 |
|  | ΔY/ΔZ | Mean | -0.45 | -0.43 | -0.45 | 97 (92.4%) | 0.037 | -0.575 | 0.650 | 0.9048 |
| Platelet | ΔX | Mean | 20.85 | 22.00 | 20.41 | 89 (84.8%) | 2.029 | -1.085 | 5.143 | 0.2015 |
|  | ΔY | Mean | 39.29 | 50.92 | 34.75 | 89 (84.8%) | 18.478 | -16.890 | 53.846 | 0.3058 |
|  | ΔY/ΔX | Mean | 2.11 | 5.14 | 0.93 | 89 (84.8%) | 5.062 | -0.741 | 10.866 | 0.0873 |
|  | ΔY/ΔZ | Mean | -0.58 | -0.74 | -0.52 | 89 (84.8%) | -0.296 | -0.764 | 0.171 | 0.2140 |
| INR | ΔX | Mean | 1.77 | 1.62 | 1.82 | 99 (94.3%) | -0.216 | -0.422 | -0.009 | 0.0404 |
|  | ΔY | Mean | 0.66 | 0.75 | 0.63 | 99 (94.3%) | 0.111 | -0.035 | 0.256 | 0.1361 |
|  | ΔY/ΔX | Mean | 0.41 | 0.49 | 0.38 | 99 (94.3%) | 0.111 | 0.017 | 0.205 | 0.0210 |
|  | ΔY/ΔZ | Mean | -0.02 | -0.03 | -0.02 | 99 (94.3%) | -0.010 | -0.027 | 0.007 | 0.2371 |

RLV indicates remnant liver volume; AST, aspartate aminotransferase; ALT, alanine aminotransferase; ALP, alkaline phosphatase; INR, international normalized ratio.

ΔX = time to peak from baseline until 2nd outpatient visit.

ΔY = peak difference from baseline until 2nd outpatient visit.

ΔY/ΔX = velocity to peak.

ΔY/ΔZ = recovery rate from peak.
